# Factors associated with client satisfaction and implementation challenges for community-based health insurance in Ethiopia: beneficiaries' perspectives

**DOI:** 10.3389/fpubh.2025.1556560

**Published:** 2025-07-16

**Authors:** Habtamu Negesse, Tarekegn Negese, Shewayirga Assalf, Abdu Oumer, Kedir Nuredin, Tewkel Reshid, Kedir Teji Roba

**Affiliations:** ^1^Japan Tobacco Enterprise, Addis Ababa, Ethiopia; ^2^Nutrition Coordination Office, Ministry of Health, Addis Ababa, Ethiopia; ^3^Department of Business, Select Business and Technology College, Addis Ababa, Ethiopia; ^4^College of Health and Medical Sciences, Haramaya University, Harar, Ethiopia; ^5^Department of Biobehavioral Health, Pennsylvania State University, University Park, Pennsylvania, PA, United States

**Keywords:** client satisfaction, CBHI, community based health insurance, implementation challenges, Ethiopia

## Abstract

**Background:**

Community-based health insurance (CBHI) is implemented in Ethiopia to enhance healthcare access and provide financial risk protection for the vulnerable people. However, it falls short in fulfilling customers' needs and could be challenged by potential constraints, needing further investigation. This study was to assess factors associated with client satisfaction and implementation challenges for CBHI in Ethiopia.

**Methods:**

A mixed-methods (quantitative and qualitative) survey was employed using face-to-face interviews combined with key informant interviews (KII) using appreciative inquiry to learn more about the practice of CBHI. The quantitative part employed structured questionnaires, and interviews were conducted among a random sample of 782 CBHI beneficiaries using the pretested and validated Ethiopian Health Insurance Agency (EHIA) tool holding multiple dimensions, to measure client satisfaction level and quality. In addition, KII was conducted with 16 key informants from program implementer and facility planning units. The data were analyzed and presented in descriptive statistics. The KII were transcribed, translated, and analyzed using descriptive narrative analysis.

**Results:**

A total of 782 CBHI beneficiaries with mean age of 47.6 (±11.6) years and 60.4% females were interviewed. With regard to dimensions of satisfaction, 74.3% reported that CBHI supported services at health facilities are accessible while 67.1% have trust in the health system and 61% reported to get quality services. Almost all were satisfied with the opening hours for service and information provided about the CBHI program. However, some respondents express dissatisfaction with the collection process and registration fee. Overall, about 32.4% (95% CI: 29.1–35.8) of the beneficiaries were satisfied with the CBHI services. CBHI implementation is at full scale in the area but its implementations are challenged by limited resource availability, administrative processes, insurance coverage, availability of essential services, and payments schemes. On the other hand, age (AOR = 3.66; 1.36–9.87), family size (AOR = 4.02; 2.13–7.59), education (AOR = 2.21; 1.34–3.66), and illness history (AOR = 3.15; 1.94–5.12) were associated with dissatisfaction with CBHI services.

**Conclusion:**

Only one-third of CBHI users were found to be satisfied with the service and it is associated with age, literacy, family size, and illness history. Key challenges cited include drug shortages, limited laboratory services, and bureaucratic policies, which should be addressed well.

## Introduction

Community-based health insurance (CBHI) has emerged as a means to improve healthcare access and provide stable income for healthcare provision. It represents a pivotal strategy in enhancing healthcare accessibility and sustainability especially the most economically disadvantaged segment of the population (1). The program aims to mitigate financial barriers to healthcare services through insured minimum health care packages, thereby improving overall health outcomes. This initiative not only provides a safety net for vulnerable populations but also foster a sense of community ownership and responsibility toward healthcare provision. Moreover, CBHI schemes contribute to the stability of healthcare financing by creating a predictable income stream for healthcare providers, which helps ensure consistent service delivery and infrastructure development (2–4). Despite having these ambitions, the success of CBHI is greatly affected by many beneficiaries, government, health care quality, access, and resource constraints limiting the wider coverage of such CBHI programs. To strengthen CBHI program to full scale requires understanding the client satisfaction, ability to meet beneficiaries expectations and ultimately realizing its full potential in promoting equitable healthcare access and sustainability (1, 5–7).

In Ethiopia, CBHI was introduced in 2010, primarily targeting the vulnerable segment. The country has implemented this social protection scheme in rural and urban areas with more enhanced initiatives in the recent years. Hence, Ethiopia has considered CBHI as a corner stone to the healthcare system to achieve universal health coverage (1, 5, 8, 9). This also allowed the country to mitigate the impoverishing effects of health expenditure in developing countries and counterbalance the increasing per capita medical expenditures, which could hinder health care seeking behaviors (3, 5, 10). The program is being implemented in several regions at a scale for maximum coverage (1, 11, 12). Hence, CBHI holds a promising strategy to enhance health care accessibility by protecting households from out-of-pocket expenditure (1, 8, 13). However, the implementation of CBHI could face a multifaceted context specific challenges and problems with satisfaction among the beneficiaries which are very crucial for its effectiveness.

For instance, previous studies have highlighted the importance of interventions such as enhancing beneficiaries' awareness, improving premium collection timing, strengthening scheme management, and enhancing service quality for the enrollment and sustainability of CBHI programs (4, 12, 14). More importantly, meeting the beneficiaries' basic health care requirements and essential expectations is very important dimension for better satisfaction and sustainability of the program. However, the achievement of CBHI programs in Ethiopia has fallen short of expectations, and there which could be partly attributed to client characteristics, quality service provision and nature of the CBHI scheme (14, 15). At the government side, fragmented contribution payment collection system, limited availability of essential health care services, and limited availability of supplies and equipment, were among the identified challenges (4, 16). However, depending on the nature of the community, implementation scale and contexts, the level of client satisfaction, and implementation challenges could vary a lot. The current study area is selected purposively due to the unique socioeconomic vulnerability among the other sub cities and also the implementation of the CBHI also targets almost all the residents. Moreover, almost all districts were implementing CBHI in this sub city and have higher proportion of eligible households for CBHI. This created a unique need to explore the factors associated with client satisfaction and implementation challenges for CBHI in the area. Hence, this research seeks to provide valuable insights and context-specific evidence for the further development and improvement of CBHI (16–18).

With all these, there are limited number of studies conducted on CBHI indicating that there is a dearth of research focusing on the client satisfaction done using robust methodology and implementation practices (20, 21). For instance, existing research on CBHI primarily focus on single dimension using quantitative methods only with further explorations using qualitative methods (14, 19–21). *Kolfe Keranio sub city* being actively implementing CBHI compared to other sub cities and given economic vulnerability of the population there, there is no study conducted to comprehensively assess CBHI implementation. Moreover, as the implementation status has variations across regions and geographic locations, it is imperative to have a context specific evidence to inform local policies and decisions on CBHI. Furthermore, the presence of unclear associations and patterns in client satisfactions and implementation challenges requires further exploration using mixed method and robust approaches. These overall implies for lack of updated and comprehensive understanding on client satisfaction and CBHI program implementation in urban settings, especially in this peculiar setting. Therefore, the current study was to identify factors associated with client satisfaction among CBHI beneficiaries and assess implementation challenges of CBHI in *Kolfe Keranio sub city* in Ethiopia.

## Methods and materials

### Study settings, design, and population

The study was conducted in *Kolfe Keranio sub city*, which is one of the 11 sub cities in Addis Ababa, the capital city of Ethiopia. This sub city was purposively selected due to the higher overall proportion of eligible households for CBHI due to prevailing low socioeconomic condition and the implementation of CBHI is almost at scale as compared to other sub cities. The total population of *Kolfe Keranio sub city* is estimated to be 617,526; 296,019 males and 321,507 females, as per the national projected population for the year 2023 (22) and it is composed of 15 administrative districts. In the selected sub city, there are four CBHI pilot districts where a total of 4,723 beneficiaries are using CBHI. *Kolfe Keranio sub city*, where a significant proportion of the population falls below the poverty line (48%) and are beneficiaries of the CBHI program (1).

### Study design and population

This cross-sectional mixed method (quantitative and qualitative) study was conducted among a purposively selected sub city of Addis Ababa (*Kolfe Keranio sub city*), Ethiopia. Hence, a descriptive qualitative design was employed among purposefully selected Key informants (KIs) among facility managers, and planning unit (district finance head, woreda health office head, woreda CBHI focal, and health center heads). As CBHI is being implemented in four pilot districts within the sub city, beneficiaries from these districts were targeted for this survey. The target population for the quantitative study was all adults aged over 18 years from CBHI beneficiary household selected randomly from the four selected districts within the sub city. Hence, those randomly selected households from each selected EAs who were using CBHI scheme were eligible for the study.

### Sample size determinations and sampling procedure

The sample size for quantitative study was determined using the single population proportion formula by assuming a proportion estimate of 58% for client satisfaction for CBHI services from the previous study (1), a 95% confidence level, a significance level of 5%, and a 5% margin of error for proportion. Hence, the sample size required for this study was 782, after considering the design effect of two, and a 10% non-response rate. A total of 782 CBHI beneficiaries were required for this study and a total of 782 were included in the study.

The sample size for the qualitative interview was determined using theoretical saturation point until information saturation. Moreover, studies depict that a minimum of 12–16 interviews could be sufficient, although this might depend on the issue under investigation (23).

A multistage sampling procedure was used to select study participants. In the primary sampling stage EA (EA were clustered by the former Central Statistics Agency) that contain a minimum of 30 members per EA. The second stage was household, where the total sample was proportionally allocated to the size within the selected EA. The complete list of households in EAs for all selected EAs was obtained, and the allocated number of samples (about 30) was included in the final recruitment. These households were selected using simple random sampling using the sampling frame obtained above. Within the selected household, the father, mother, or any old adult family member aged above 18 years were selected and interviewed. While the purposive sampling techniques were employed for KII from district or woreda finance head, woreda health office head, woreda CBHI focal, and health center heads. Hence, a total of 151, 210, 225, and 196 CBHI member households were selected randomly from district 4, 5, 8, and 9 to be included in the current study. These numbers (quota) were allocated for each district (four per each district) with a total of 16 key informants.

#### Data collection methods

A quantitative data was collected using a pretested questionnaire via face-to-face interviews using a structured questionnaire. The tool contains Likert scale-type questions with options ranging from strongly disagree to strongly agree to the mentioned items. Data were collected from selected households using the Ethiopian Health Insurance Agency (EHIA) questionnaire adopted from previous literature. The tool was obtained from EHIA which is validated and it has multiple domains including perceived quality of services, health seeking behavior, satisfaction with CBHI services, implementation challenges, and barriers. As the tool was validated in Ethiopian context, we have adopted it and a pilot test was conducted before the survey for validation and amendment (1, 24, 25). The data was captured via the Open Data Kit (ODK) mobile application. Five data collectors to collect structured questionnaires under the close supervision of the principal investigator and supervisor.

Likewise, key informant interview (KII) guided questions were developed for district or woreda finance head, woreda health office head, woreda CBHI focal, and health center heads. Hence, a total of 16 KIIs were collected from four woredas (four samples from each district). The participants were district or woreda finance head, woreda health office head, woreda CBHI focal, and health center heads. The KIIs were collected by experienced degree graduates in health science, where probing and other interview techniques were employed. Field notes and audio transcripts were recorded with full written, informed consent. Five data collectors with diploma holders with qualifications in health and health technicians were deployed for 10 days.

### Variables of the study

The dependent variables of the study were client satisfaction with CBHI services considering the beneficiary perspective on implementation status and implementation challenges of CBHI. Although the implementation status of CBHI has multiple dimensions, we have focused our investigation on beneficiaries' perspective captured using a more robust satisfaction measures from multiple dimensions. For the CBHI implementation status, we have used the CBHI practice and challenges and a CBHI service quality dimension of the EHIA questionnaire was applied. The independent variables were sociodemographic characteristics (age, sex, family size, educational status, occupation, history of illness, admission, perceived quality of health services, health seeking behaviors, and income of the household.

Moreover, client satisfaction was assessed using the client satisfaction module of the EHIA questionnaire using Likert scale. Those individuals who score above 70 percentile of the satisfaction score were classified as being satisfied with CBHI service. In addition, perceived CBHI service quality was measured using a Likert scale in which quality relevant questions were included in the module.

### Data quality assurance

To assess the reliability of the instrument, we employed a standardized and informed data collection procedure for each respondent. In addition, a reliability analysis using Cronbach's alpha was conducted for each major construct. Overall, the item reliability of the tool was found to be above 0.70 for each of the three constructs, which indicates good inter-item reliability. Hence, high reliability was observed for the practice and challenges construct (27 items; Cronbach's alpha = 0.79), CBHI service quality (6 items; Cronbach's alpha = 0.72), and CBHI service satisfaction (9 items; Cronbach's alpha = 0.91).

The respondents were informed of the purpose of the interview and of the need to respond truthfully. To ensure the reliability of the measurement instrument, the tool was standardized across interviews. The research instrument was tested for content validity by giving the questionnaire to the supervisors and CBHI experts in the district. Then, the tool was piloted in one district other than those selected for this study yet having CBHI scheme within the district. Also, the data collection was performed strictly following the procedure, and we employed controlled data collection using the ODK mobile data collection system.

Moreover, training was given for data collectors and supervisor on the overall data collection process, tool, procedure, consenting, and ODK mobile data collection. This was followed by a pretest on 20 households where necessary amendments were made before actual data collection. The overall process of data collection was monitored by the investigators and supervisors. The ODK tool allows for enhanced data quality through controlled and validated data entry mechanisms. To assure data quality, relevant skipping rules and validation rules were applied accordingly.

### Methods of data analysis

The collected data was cleaned, checked for consistency, and entered into SPSS version 24 for analysis. The data was presented in statistical graphs, charts, frequencies, and percentages. In addition, the mean and standard deviation were reported accordingly. Likert scale questions on knowledge, attitude, and beneficiary satisfaction with the CBHI scheme were summarized, and summary scores were calculated. The results are presented in statistical tables and figures as appropriate. The reliability of the items for these constructs was calculated using Cronbach's alpha.

Moreover, a 70 percentile was used to classify beneficiaries' attitude and satisfaction as poor or good (below the 70 percentile score) and satisfied or dissatisfied by the CBHI services. The same method was also applied to measure perceived quality of services in health facilities. Statistical significance was declared at a *p-value* below 0.05. A stepwise backward logistic regression was employed after checking multicollinearity and confounding. Adjusted odds ratio was reported along with the 95% confidence interval. Biologically plausible factors associated with CBHI clients' satisfaction were considered for the multivariable model. In addition, factors associated with satisfaction in bivariable analysis at with *p-value* below 0.25 were considered as candidate for the multivariable model. The adjusted odds ration along with 95% confidence interval was reported.

For the qualitative data, the audio transcripts were transcribed and translated to English language and a descriptive content analysis was conducted to identify the main themes. The findings were summarized in narrative descriptions based on the identified major contents. These are presented in descriptive statements supported by quotes and interpretations.

### Ethical considerations

This study was ethically approved by Addis Ababa Health Bureau Ethics Committee. Informed consent was obtained from each study participants for KII as well after the detailed study procedures was explained to the study participants in local language. The study was strictly was conducted on voluntary basis. The study was conducted in accordance with Helsinki declaration. Interview was conducted in private area and no personal identifiers were collected. The collected data is kept confidentially with the investigator and will not be shared.

## Results

### Socio-demographic information

In this study, a total of 782 CBHI beneficiaries were interviewed, with a response rate of 100%. The mean (standard deviation) age of the study participants was 47.6 (±11.6) years, with a range of 22 to 86 years of age. About 81.2% are between the ages of 15 and 65, while 18.8% of them are over 65 years old. More than half of the respondents were female, accounting for 60.6%. About 43% and 35.2% of respondents reported having a monthly income level below 2,000 and 2,000–3,500 ETB, respectively. Concerning the marital status of respondents, the highest proportions of respondents were married (66.5%). Moreover, the largest proportion of the study participants were merchants (26.3%), and private employees (24.3%). About 32.0% of the respondents attended primary education. Concerning the average family size, 57.8% had 1–5, while 42.2% had below (Table 1).

**Table 1:** Socio demographic characteristics of CBHI beneficiaries in Addis Ababa, Ethiopia.

**Variables**	**Frequency (%)**	
Age categories	15–65 years	635 (81.2%)
	Above 65 years	147 (18.8%)
Sex	Male	308 (39.4%)
	Female	474 (60.6%)
Family size	1–5	452 (57.8%)
	Above 5	330 (42.2%)
Religion	Orthodox	398 (50.9%)
	Muslim	237 (30.3%)
	Protestant	109 (13.9%)
	Catholic	38 (4.90%)
Educational status	No formal education	193 (24.7%)
	Primary education	250 (32.0%)
	Secondary education	185 (23.7%)
	College and above	154 (19.7%)
Occupation	Government	133 (17%)
	Private employ	190 (24.3%)
	Merchant	206 (26.3%)
	Farmer	4 (0.50%)
	Daily laborer	130 (16.6%)
Marital status	Single	77 (9.80%)
	Married	520 (66.5%)
	Divorced	81 (10.4%)
	Widowed	104 (13.3%)
Monthly income in Ethiopian Birr	0–2,000	336 (43.0%)
	2,001–3,500	275 (35.2%)
	3,501–5,000	153 (19.6%)
	5,001–6,500	17 (2.20%)
	Above 6,501	1 (0.10%)

### Health seeking behaviors of respondents

Concerning health-seeking behaviors, 76.5% of the respondents reported to visit either government and/or private health facilities when they get sick. However, 10.1%, 8.10%, and 5.40% reported going for self-treatment at home, taking herbal medicines, and going for religious healing, respectively. Concerning the usual frequency of annual health care visit, 58% reported once a year while 26.2% visited at least four times a year. Health care seeking behavior is quite high; 84.9 percent agreed that they would visit health centers when they get sick, 11.6% of the respondents prefer to visit hospitals, and only 3.5% choose to visit other health institutions. Less than half of the respondents (45.3%) responded that they have visited health centers once a year, 28.4% responded twice a year, 14.2% claimed three times a year, and the remaining 6.5%, 2.7%, and 2.9% of the respondents have visited health institutions about four, five, and more than five times, respectively.

### Perceived quality of health services related to CBHI

More than half of the respondents (67.1%) have trust in the health system, and 61% of respondents agreed that the service they have received at a health facility was perceived as quality. A large number of households experience delays in receiving immediate healthcare at health centers, with wait times ranging from 4–12 h for the majority of members. Only a few members reported wait times of 1–3 h, highlighting the long waiting periods for health services. Moreover, 29.8%, 59.6%, and 65.1% of the respondents agreed that they get immediate care at health facilities with due respect and in a friendly manner, respectively. A large number of households experience delays in receiving immediate healthcare at health centers, with wait times ranging from 1–12 h for the majority of members. Only a few members reported wait times of 1–3 h, highlighting the long waiting periods for health services.

**Figure 1:**
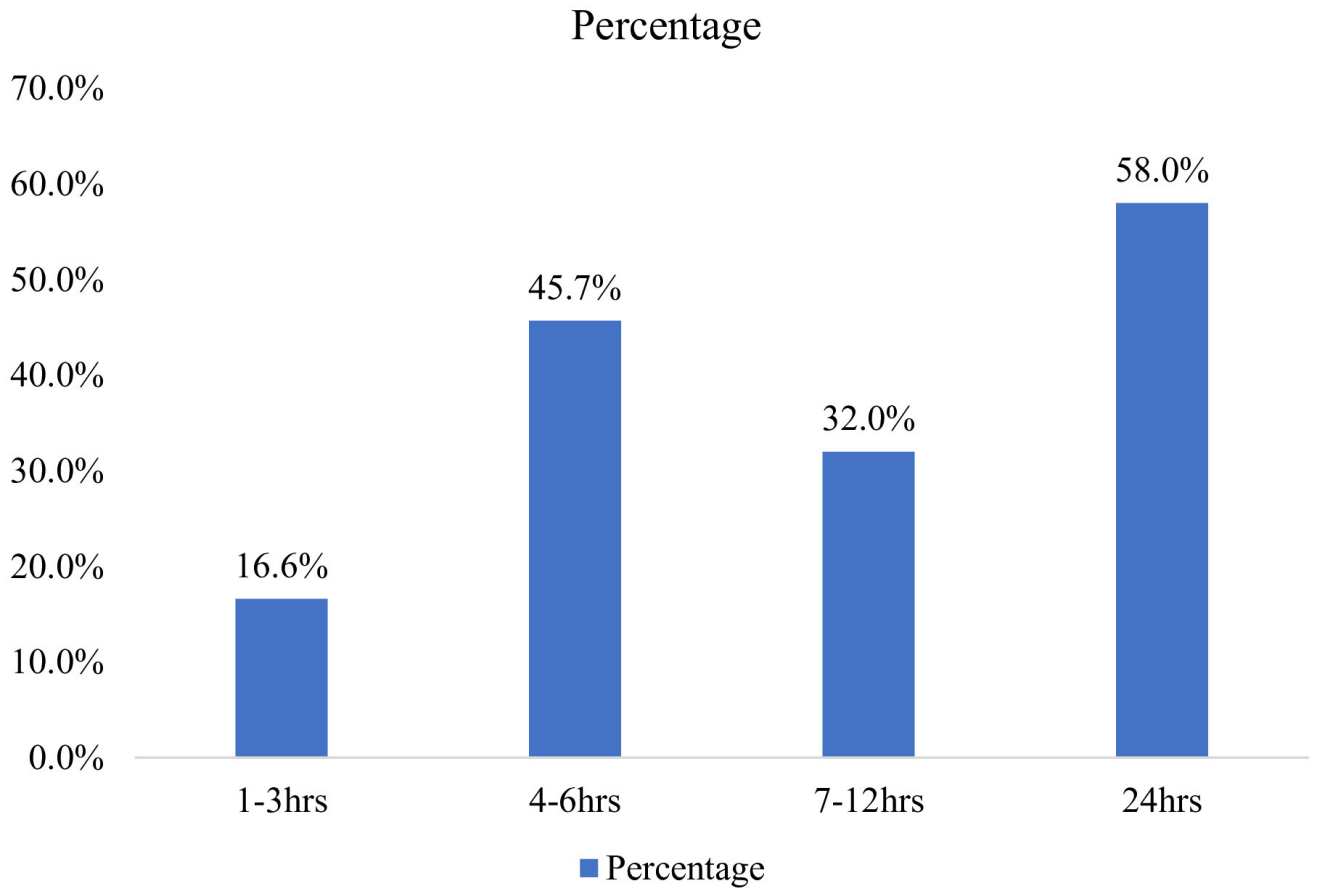
Length of stay at health facility for health services under CBHI scheme in Addis Ababa, Ethiopia.

To get health care service, 16.6% reported that it takes 1–3 h while significant proportion of the clients reported that it took 1 day (Figure 1).

### CBHI service satisfaction of respondents

About 38.8% and 10.4% agreed and strongly agreed with the suitability of the CBHI opening hours, while 32% disagreed. However, 15.8% of respondents were dissatisfied with the collection process for insurance cards. Regarding the collection of insurance cards and the service after paying the registration fee, more than half of the respondents are satisfied, while a few members have concerns about the collection process and the service received. In addition, 48.5% agreed and 17.3% strongly agreed about the registration fee for CBHI before enrollment, but 15.7% disagreed on the presence of a registration fee before enrollment. On the other hand, 35.7% of the respondents were happy with the schedule for paying the premium. Also, 69.7% of the respondents agreed that they were satisfied with the information provided about CBHI, and 64.3% of the respondents were satisfied with CBHI packages. About 60.1% of them agreed and 16.9% strongly agreed with the statement that they needed to stay enrolled in the CBHI scheme. Finally, more than half of the respondents (53.7%) agreed that they needed CBHI to be scaled up to other districts, but only 0.9% of them disagreed. Overall, 32.4% of the beneficiaries were satisfied with the CBHI services, considering 70 percentiles and above for good satisfaction as the cutoff point (Table 2).

**Table 2:** Descriptive statistics on the quality of health services under CBHI scheme.

**Dimensions of satisfaction**	**Strongly agree**	**Agree**	**Neutral**	**Disagree**	**Strongly disagree**
Household members are satisfied with the opening hours of the CBHI	81 (10.4%)	303 (38.8%)	161 (20.6%)	217 (27.7%)	43 (5.5%)
Household members are satisfied with the collection process of insurance cards	62 (7.9%)	407 (52%)	190 (24.3%)	99 (12.7%)	24 (3.1%)
Household members are satisfied with the time to make use of the CBHI program after payment of registration fee	135 (17.3%)	379 (48.5%)	145 (18.5%)	102 (13%)	21 (2.7%)
Household members are satisfied with the schedule for paying of premium	44 (5.6%)	279 (35.7%)	192 (24.6%)	215 (27.5%)	43 (5.5%)
Local CBHI management is trust worthy	26 (3.3%)	266 (34%)	405 (51.8%)	66 (8.4%)	19 (2.4%)
Household members are satisfied with the information provided about CBHI	67 (8.6%)	476 (60.9%)	153 (19.6%)	71 (9.1%)	15 (1.9%)
Household members satisfied with CBHI packages	63 (8.1%)	440 (56.3%)	171 (21.9%)	75 (9.6%)	17 (2.2%)
Household members stay enrolled in the CBHI scheme	132 (16.9%)	470 (60.1%)	102 (13%)	64 (8.2%)	14 (1.8%)
CBHI need to be scale up to other districts	141 (18%)	420 (53.7%)	187 (23.9%)	27 (3.5%)	7 (0.9%)
**Client satisfaction level**					
Good	253	32.4%			
Poor	529	67.6%			
Average scored (SD)		22.4 (6.31)			

### Practice and challenges related to CBHI implementation

**Figure 2:**
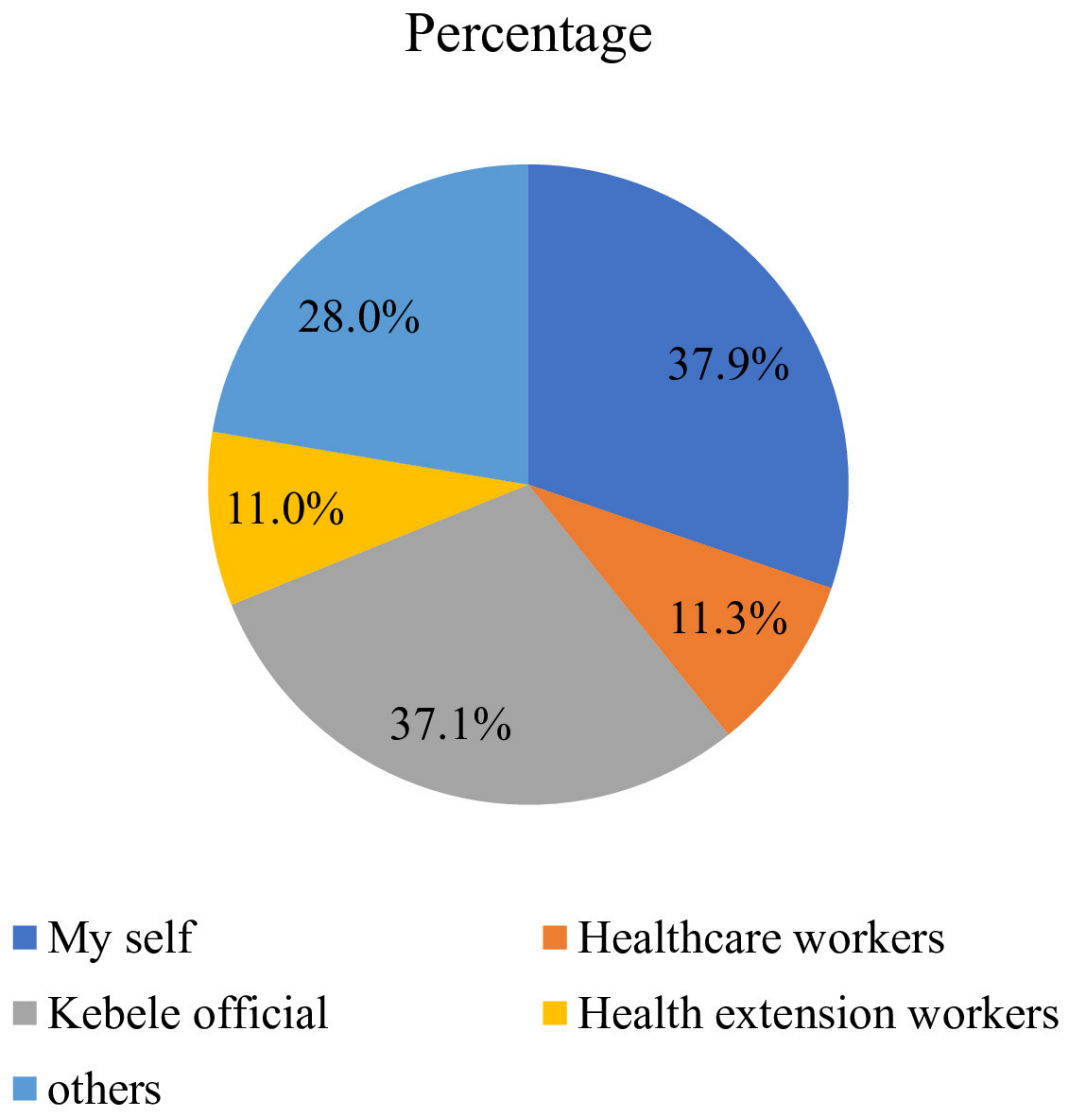
Showing the initiator of CBHI membership of beneficiary households in Addis Ababa, Ethiopia.

About 37.9% and 37.1% of households affirmed that their enrollment in CBHI membership was initiated by themselves and Kebele administrators, respectively. The remaining 22.3% was initiated by health professionals. About 36.3% of the respondents were enrolled in CBHI for 1–2 years, and 7.0% had a long period of membership in the program (more than 7 years). Meanwhile, 33.2% stated that their enrollment didn't exceed 1 year. Also, only 30.2% are founded under the category of indigent CBHI scheme type (Figure 2).

A higher number of the respondents (66.5%) did not have a history of previous illnesses, while 66.0% of the respondents reported that they had never been admitted to the hospital. The majority (83.5%) reported having anticipated worries about paying for health care services when getting sick, indicating the huge perceived costs of health care. On the other hand, 72.4% of them mentioned that they have renewed their CBHI card, and 28.9% of them were dissatisfied with the equality of health care services without patients (Table 3).

**Table 3:** The perceived CBHI services and coverages among adult CBHI beneficiaries in Addis Ababa, Ethiopia.

**Constructs**	**Yes; freq (%)**
Do you have a history of previous illness?	261 (33.4%)
Have you ever been admitted to the hospital?	266 (34%)
Do you worry about paying when get sick?	653 (83.5%)
Have you renewed your ID for this year (2013)?	566 (72.4%)
Are you getting equal treatment with those of OOP	556 (71.1%)
Are you happy with the services of health institutions?	495 (63.3%)
During the recent visit to the health care institutions, did the sick family members receive drugs?	404 (51.7%)
During the recent visit to the health care institutions, do you think that the sick family members received the correct prescribed drug?	418 (53.5%)
During the recent visit to the health care institutions, did the sick family members receive laboratory services?	452 (57.8%)
During the recent visit to the health care institutions, do you think that the sick family members received the required laboratory services?	473 (60.5%)
Have you ever participated CBHI related meeting for last 3 months?	232 (29.7%)
CBHI is good way of helping clients to relive health expenditure	643 (82.2%)
CBHI covers only care from public health institutions	381 (48.7%)
CBHI covers only care with in the country	753 (96.3%)
CBHI doesn't cover transportation fee	588 (75.2%)
CBHI covers inpatient care	572 (73.1%)
CBHI covers outpatient care	617 (78.9%)
CBHI will no cover medical care for cosmetic values	760 (97.2%)

The higher number of respondents (63.3%) agreed that they are happy with the services of health institutions. Moreover, about 51.7% of the CBHI member families have received drugs during their recent visits to the health care institutions. About 53.5% of the respondents mentioned that sick family members had received the correct prescribed drug during their recent visit to the health care institutions, while 46.5% responded negatively. About 42.2% of the respondents mentioned that they did not get laboratory service during their recent visit to a health institution. Respondents were also asked whether the CBHI scheme is a good way of helping clients relieves health expenditures; about 17.8% of them disagreed. Less than fifty percent (48.7%) of the respondents agreed that the CBHI scheme covers only care from public health institutions and 96.3% for country expenses only (Table 4).

**Table 4:** Factors associated with client dissatisfaction with the CBHI service among CBHI beneficiaries in Addis Ababa, Ethiopia.

**Factors**	**Categories**	**CBHI client satisfaction**		**aOR**	***P-value***
		**Dissatisfied**	**Satisfied**		
Age category	age between 15 to 65	491 (67.0%)	242 (33.0%)	1	
	Age >65	38 (80.9%)	9 (19.1%)	3.66 (1.36–9.87)	0.010^*^
Gender	Male	220 (71.4%)	88(28.6%)	1.39 (0.93–2.08)	0.113
	Female	309 (65.2%)	165 (34.8%)	1	
Occupation	Government	95 (72.0%)	37 (28.0%)	1	
	private	124 (65.3%)	66 (34.7%)	1.03 (0.55–1.93)	0.925
	Merchant	125 (60.7%)	81 (39.3%)	0.73 (0.40–1.34)	0.309
	Unemployed	185 (72.8%)	69 (27.2%)	1.46 (0.77–2.77)	0.250
Educational status	Illiterate	113 (58.5%)	80 (41.5%)	1	
	Literate	416 (70.6%)	173 (29.4%)	2.21 (1.34–3.66)	0.002^*^
Marital status	Married	345 (66.3%)	175 (33.7%)	1	
	Unmarried	184 (70.2%)	78 (29.8%)	1.43 (0.94–2.18)	0.095
Family size	< 5	424 (64.8%)	230 (35.2%)	1	
	≥5	105 (82.0%)	23 (18.0%)	4.02 (2.13–7.59)	< 0.0001^*^
History of previous illness	No	383 (73.7%)	137 (26.3%)	3.15 (1.94–5.12)	< 0.0001^*^
	Yes	146 (55.7%)	116 (44.3%)	1	
Previous admission to hospital	No	364 (70.5%)	152 (29.5%)	1.29 (0.80–2.07)	0.301
	Yes	165 (62.0%)	101 (38.0%)	1	

Statistically significant at p-value below 0.05 (^*^).

Analysis reveals that most respondents with a previous illness prefer visiting doctors over self-treatment, herbal medicine, or religious remedies, indicating a shift toward modern medical institutions. The research emphasizes the need for medical care once a year for most members, while others require more frequent visits to health centers. Over 80% of members express concerns about healthcare costs, indicating that the CBHI program targets individuals unable to afford healthcare. Notably, a significant proportion of respondents enrolled in the past 3 years, highlighting a growing demand for health services and enrollment in the CBHI program.

Challenges arise from members not renewing their membership IDs, indicating their inability to pay the premium. Also, the majority of members are satisfied with the service provided by health institutions, but there is still limited access to drugs and laboratory services. Additionally, a significant portion of CBHI members have not actively participated in program-related meetings, indicating a lack of opportunities for member input. Most respondents prefer visiting public health centers over hospitals, with limited preference for health centers. The CBHI program primarily covers domestic health services within the country and includes both inpatient and outpatient care, but it does not extend coverage to healthcare outside the country, which could be considered a constraint.

### Factors associated with satisfaction with CBHI

A stepwise backward regression was conducted for factors associated with client satisfaction with CBHI services. A total of eight variables namely; age, gender, occupation, educational status, marital status, family size, history of previous illness, and admission were important factors associated with client satisfaction. Hence, old age (aOR = 3.66; 95% CI: 1.36–9.87) and male customers (aOR = 1.39; 95% CI: 0.93–2.08) were highly dissatisfied with the CBHI services compared to counter parts. CBHI clients with extended family size (aOR = 4.02; 95% CI: 2.13–7.59), better literacy (AOR = 2.21; 95% CI: 1.34–3.66) were more likely to be dissatisfied by the CBHI services and implementations compared to the counter parts. Those CBHI users without history of previous illness (aOR = 3.15; 95% CI: 1.94–5.12) and without history of previous admissions (aOR = 1.29; 95% CI: 0.80–2.07) were more likely to be dissatisfied with the CBHI services as compared to those without previous history (Table 4).

### Qualitative study findings

The interviews with key informants reveal that the CBHI package has had positive effects, with increased utilization of health facilities and satisfaction with health providers' treatment and respect during service delivery. However, challenges arise from low member attitudes, a lack of drugs in health centers, and difficulties in renewing ID cards and paying premiums on time. Ethical issues are also identified, such as giving priority to latecomers through financial incentives and bureaucratic challenges in health centers. A 32 years old male participant dictated that “*…there is somehow ethical related problems i.e., during the delivery of health services priority was given to late comer by giving money to security persons and card room workers.”* This emphasizes that such challenge during CBHI implementation, leading to dissatisfaction among clients regarding health providers' services.

The introduction of the CBHI package has provided opportunities for household members to increase their health awareness and willingness to seek healthcare, leading to a rise in program enrollment. Key informants have also dictated that the policy offers free health services to indigent members, demonstrating the government's commitment to delivering healthcare effectively even through community resource mobilizations. The CBHI program covers both inpatient and outpatient services, ensuring access to health insurance at all levels. The findings reveal that satisfaction with the quality of health services and trust in the CBHI package contributes to increased utilization of health facilities. One participant (female, 25 years old) mentioned that “… some malpractices among health professionals coupled with more administrative process for CBHI usually creates a challenge for beneficiaries….” Moreover, the collections of insurance cards and service after paying the registration fee are seen as positive aspects of the program. A 42 years old male participant stated that “… in the district, the cards are distributed on time to the beneficiaries helping beneficiaries to use their CBHI schemes for their health expenditure.”

However, the CBHI program faces several challenges that hinder its effectiveness. Shortages of drugs and laboratory services, as well as the inability to cover household transformation fees and related expenses, are major obstacles. A 36 years old female participant indicated that “… shortage of supplies (laboratory reagents, medical supplies, imaging) with the health centers usually makes difficult to the beneficiaries to get the required medications and services. This exposed them to unnecessary out of pocket expenditures… creating dissatisfaction.” Furthermore, the low attitude of members toward premium payments and instances of bribery from security guards and card room workers, along with bureaucratic issues during the health insurance process, lead to dissatisfaction among household members. This supported by participants saying

“*Bureaucracy in health centers at the time of CBHI policy implementation make it difficult to the household members to get the necessary health services on time (male, 43 years old).”*

## Discussion

The aim of this study was to identify factors associated with satisfaction with CBHI services and implementation challenges of CBHI in *Kolfe Keranio sub city*, Ethiopia. The findings showed that overall, 32.4% of the CBHI beneficiaries were satisfied with the overall CBHI services and old age (aOR = 3.66), extended family size (aOR = 4.02), better literacy (aOR = 2.21), and without history of previous illness (aOR = 3.15) were significantly associated with client satisfaction with CBHI services. Moreover, there are problems associated with longer waiting times to get service, management delays with the CBHI system, and limited availability of essential drugs and laboratory facilities. Although the majority of household members are satisfied with the opening hours and information provided about the CBHI program, respondents reported dissatisfaction with the collection and registration fee. Though it is a recently developed health policy, CBHI in Ethiopia has now been regarded as a good means to provide health care services in major regional states of the country, including the city of Addis Ababa (6, 17, 26). On the other hand, the current level of client satisfaction is low, which could affect the effectiveness of the program. It is imperative to monitor and improve governmental health services while designing effective CBHI policy.

Beneficiaries perceived that there are improvements in having a better health care service with the CBHI enrollment. This could be influenced by awareness level, quality health care services, and affordability of the payment (27, 28). A report on final CBHI pilot schemes in Ethiopia depicted that community awareness would help in increasing enrollment of all eligible individuals or households (1). Hence, continuous monitoring and quality improvement scales could help sustain CBHI. On the other hand, problems related to premium payment for the most vulnerable population shall be covered by other social protections mechanism (29, 30). Qualitative findings with theme of “*… availability of essential services and the quality of health care at health facility…”* is crucial determinants of client satisfaction with CBHI services.

The other critical challenge is related to limited drugs and availability of laboratory services, in governmental health facilities. Other similar study (31) has also indicated that the lack of drugs and supplies in the health center pharmacies was the major challenge faced by beneficiaries. This further exposes them to excess unplanned out of pocket expenditures. Qualitative finding showed that “*... shortage of supplies (laboratory reagents, medical supplies, imaging) with the health centers usually makes difficult to the beneficiaries to get the required medications and services.”* These are highly related to client satisfaction with CBHI and would determine their decision to stay within the program. Hence, these contract services such as pharmacy services, laboratory facilities, and other outpatient services, shall be well equipped with the necessary resources for better quality enhancement (4, 6). Other studies also emphasized the presence of a shortage of equipment, particularly laboratory services, in clinics and public health centers (27–30), which could be a major point for client dissatisfaction. Furthermore, there is a need to establish a functional and reliable feedback system from beneficiaries to plan for improvement (13).

Regarding premium and service fees, the study found that many household members were unable to afford and expressed dissatisfaction with the payment schedule under the CBHI scheme. One key informant also showed that “*since some of the beneficiaries re unable to pay for their insurance even though it is small, we usually create a mechanism to cover this through resource mobilizations.”* In addition, some members often obtain their CBHI identity cards when they fall ill (1, 12, 16). Hence, the issue of payment affordability is a significant concern for many households, particularly because many CBHI policy members in the target area are impoverished. However, these findings contrast with another similar study where more than 91% of households did not complain about the premium being too high (17, 32, 33), which could be related to difference in the purchasing power of the beneficiaries. The other potential implementation challenge is the quality of services and trust in health care. Trust in the CBHI policy plays a role in members' ease of enrollment and their confidence in seeking treatment at health institutions (19, 34). For instance, other study indicated that many household members have a positive perception of and trust in the healthcare services provided under CBHI (21). This contributes to satisfaction with CBHI services, and fosters a strong relationship between policy providers and beneficiary households. Previous studies have also highlighted the benefits of trust, including improved communication, adherence to medical advice, better health outcomes, and a positive patient experience (21, 35). Furthermore, this can significantly influence members' decision to remain enrolled in CBHI program (3, 19, 21, 36).

Client satisfaction is a crucial factor in determining the success of healthcare facilities under the CBHI policy. Service dissatisfaction is very crucial for clients to stay within the CBHI program (14, 17, 20). This also encompasses the service provided by health care professionals in ethical and compassionate way building a confidence among beneficiary households. Hence, adherence to high standards of work performance is needed for service quality and CBHI beneficiaries' satisfaction (3, 12, 18). A similar study (31), suggests that the high satisfaction rate observed here may encourage the scaling up of the CBHI scheme and service quality in rural areas, as CBHI has been shown to increase healthcare care access and utilization than ever.

Several factors are significantly associated with client satisfaction with CBHI services. Individuals in the older age group are 3.66 times more likely to be dissatisfied with CBHI services compared to younger individuals. This suggests that older individuals may have more negative experiences or have perceive greater expectations of service quality. This could also be associated with a reliance on CBH with limited opportunity to private health care or out of pocket expenditures (31).

Clients from larger extended families and with better literacy are more likely to be satisfied with CBHI services. This finding implies that having a larger support network within the extended family may positively influence the perception of CBHI services. Furthermore, higher literacy may contribute to a better understanding and utilization of the services offered by CBHI. This could lead to higher satisfaction. Individuals without a history of previous illness are 3.15 times more likely to report dissatisfaction with CBHI services compared to those with a history of illness. This finding implies that individuals who have not experienced significant health issues in the past may have limited exposure to the services, resulting in higher expectations (31). On the other hand, individuals with a history of illness may have a more positive perception of the services provided by CBHI. Overall, the results indicate that old age, larger extended family size, better literacy, and no previous history of illness are significant factors associated with higher client dissatisfaction with CBHI services. Other study also emphasized that CBHI process- and management-related problems and poor laboratory services are associated with satisfaction (24). Overall, these findings provide insights into the factors that contribute to a positive perception of CBHI and can help inform strategies to improve client satisfaction and overall healthcare delivery within the CBHI system.

However, the findings of the current study should be interpreted in the light of some limitations. The year in which the currents study conducted is little bit outdated where these challenges could have been improved with wider implementation of CBHI. Moreover, conducting the KII with the facility personnel could be misleading in clearly understanding the implementation challenges of CBHI. It was imperative to include in depth interview with CBHI beneficiaries as well. Moreover, the generalizability of the current study is limited to the selected sub city. Moreover, participant may to report positively (social desirability bias) and may get stuck in recalling some of their experiences. Despite these, the currents study provides a valuable input for policy decisions in the sub city and beyond.

## Conclusions and recommendations

CBHI program is effective means of improving service accessibility and utilization among the most deserving community members. This study assessed the client satisfaction and implementation challenges of CBHI program in *Kolfe Keranio sub city*, Ethiopia. The implementation of CBHI is at good stand yet practical challenges related to service accessibility, inconsistent enrollment schemes, problems with the payment, limited drug and laboratory service availability, and bureaucratic issues at still major bottle necks for client dissatisfaction. Moreover, member's satisfaction with the overall CBHI service is low and associated with age, income, educational status, illness history, and family size. This is associated with the perceived quality of services and essential service availability. Hence, addressing these potential challenges at different levels is crucial for the sustained impacts of the CBHI program. Establishing a continuous feedback mechanism from community members on service quality, customer satisfaction and CBHI implementation is crucial. More importantly, a convenient payment schemes and mechanisms, increasing health care infrastructure, availability of essential medicine and services is crucial for better CBHI implementation and client satisfaction.

## Data Availability

The original contributions presented in the study are included in the article/supplementary material, further inquiries can be directed to the corresponding author.
